# A biomarker panel to discriminate between systemic inflammatory response syndrome and sepsis and sepsis severity

**DOI:** 10.4103/0974-2700.58666

**Published:** 2010

**Authors:** Chamindie Punyadeera, E Marion Schneider, Dave Schaffer, Hsin-Yun Hsu, Thomas O Joos, Fabian Kriebel, Manfred Weiss, Wim FJ Verhaegh

**Affiliations:** Department of Molecular Diagnostics, Philips Research, High Tech Campus 12a, 5656 AE Eindhoven, The Netherlands, Germany; 1 Experimental Anesthesiology, University Hospital Ulm, Steinhoevelstr. 9, 89075 Ulm, Germany; 2 Philips Research North America, 345 Scarborough Road, Briarcliff Manor, NY 10510, USA, Germany; 3 Natural and Medical Sciences Institute, University of Tuebingen, Markwiesenstr. 55, 72770 Reutlingen, Germany; 4 Clinical Anesthesiology, University Hospital Ulm, Steinhoevelstr. 9, 89075 Ulm, Germany

**Keywords:** Biomarker, cellular mechanism, sepsis outcome, SIRS, sepsis stage

## Abstract

**Introduction:**

In this study, we report on initial efforts to discover putative biomarkers for differential diagnosis of a systemic inflammatory response syndrome (SIRS) versus sepsis; and different stages of sepsis. In addition, we also investigated whether there are proteins that can discriminate between patients who survived sepsis from those who did not.

**Materials and Methods:**

Our study group consisted of 16 patients, of which 6 died and 10 survived. We daily measured 28 plasma proteins, for the whole stay of the patients in the ICU.

**Results:**

We observed that metalloproteinases and sE-selectin play a role in the distinction between SIRS and sepsis, and that IL-1α, IP-10, sTNF-R2 and sFas appear to be indicative for the progression from sepsis to septic shock. A combined measurement of MMP-3, -10, IL-1α, IP-10, sIL-2R, sFas, sTNF-R1, sRAGE, GM-CSF, IL-1β and Eotaxin allows for a good separation of patients that survived from those that died (mortality prediction with a sensitivity of 79% and specificity of 86%). Correlation analysis suggests a novel interaction between IL-1α and IP-10.

**Conclusion:**

The marker panel is ready to be verified in a validation study with or without therapeutic intervention

## INTRODUCTION

A consensus conference in 1992 first published agreed-upon definitions of systemic inflammatory response syndrome (SIRS), sepsis (SIRS with known or suspected infection), severe sepsis and septic shock.[1] With these operational definitions in hand, statistics on incidents have been collected and have established that sepsis is the tenth leading cause of death in the USA with over 700 000 cases a year and with mortality rates above 30%.[2] The incidents continue to increase, with unacceptably high mortality rates, despite the use of a wide variety of therapies and continuing research.

During the onset of sepsis, a massive inflammatory reaction occurs in the initial phase, which is followed by a variable anti-inflammatory response.[3] These events involve chemical mediators, such as cytokines and chemokines, and inflammatory cells, such as the polymorphonuclear neutrophils and macrophages. Therefore, inflammatory as well as anti-inflammatory biomarkers could potentially be used for diagnosis and outcome prediction. Moreover, such biomarkers may stimulate evidence-based medicine, the design of preventive clinical approaches as well as personalized treatment protocols. As our first effort in this direction, we analyzed a dataset consisting of 28 plasma proteins collected from patients during their stay in an intensive care unit (ICU). The following clinically relevant aspects were addressed:

- differential diagnosis of SIRS versus sepsis;
- sepsis disease severity and
- the outcome (survival or death due to sepsis).

In this study, we demonstrate the relevance of MMP-1, -2, -7, -13 and sE-selectin for the distinction between SIRS and sepsis. The down modulation of MMPs may be relevant to facilitate receptor re-expression, which was impaired in an infection-independent systemic inflammation (SIRS). Further, we identified a set of biomarkers related to sepsis disease severity. These were IL-1α related to apoptosis and inflammation, IP-10 related to leukocyte recruitment into inflamed organs and sTNF-R2 and sFas related to a pro-inflammatory environment with little beneficial effect on functional modulation of the sepsis scenario.

## MATERIALS AND METHODS

### Patient population

Sixteen ICU patients were included in this prospective study and recorded daily over 4–15 days. All patients were surgical patients: polytraumatized, after abdominal, lung and great vessel surgery. From admission to ICU to discharge from the ICU, we aggregated clinical data into the following scores:

- SAPS II (Simplified Acute Physiological Score II): 17 variables (12 physiological variables, age, admission for medical or scheduled/unscheduled surgery and underlying diagnosis (acquired immunodeficiency syndrome, metastatic cancer, hematologic malignancy)).[4]
- SOFA (Sequential Organ Failure Assessment) score: status of six organ systems (respiration, coagulation, liver, cardiovascular, central nervous system, renal).[5]
- Sepsis score: graded, based on the international sepsis definitions, as: (1) local infection, (2) bacteremia, (3) SIRS, (4) sepsis, (5) severe sepsis, (6) septic shock.[1]

Sepsis was defined using the original 1992 ACCP/SCCM sepsis definitions.[1] In the present study, due to the 1992 definitions, a systemic inflammatory response syndrome (SIRS) was manifested in patients by two or more of the four conditions: temperature >38°C or <36°C, heart rate >90/min, respiratory rate >20/min or PaCO_2_ <32 torr and white blood cell count (WBC) >12 000 cells/mm^3^ or <4000 cells/mm^3^. If SIRS was due to a documented infection, patients were classified as sepsis patients. Severe sepsis was defined as sepsis plus organ dysfunction. Septic shock was defined as severe sepsis plus shock. Severity of sepsis is proposed to increase firstly by association with organ dysfunctions and secondly by additional shock. As recommended, in the present study, applying the 1992 sepsis definitions, organ failure was regarded to be present if patients had lactic acidosis or oliguria, or reached greater than two points in one organ system (lung, coagulation, liver, kidney) using the SOFA score.[5] Greater than two points are reached in the SOFA score for the organ system lung with PaO_2_/FiO_2_ ≤200 with respiratory support, for coagulation with platelets ≤50 000/μl, for liver with bilirubin >6 mg/dl or 102 μmol/l, for kidney with creatinine >3.5 mg/dl or >300 μmol/l or with urine output <500 ml/day. Septic shock was defined as hypotension despite adequate volume resuscitation, a systolic blood pressure of ≤90 mmHg or the need of vasopressors to keep blood pressure >90 mmHg.[1]

In total, 118 samples were collected from the abovementioned 16 patients. Six of these patients died, and 10 survived. In addition to these 16 patients, four healthy volunteers of the same age without SIRS or sepsis were included in the study, each contributing one plasma sample.

In the plasma samples, 28 biomolecules were analysed using the Luminex system (USA): 10 cytokines (TNF-α, IL-1β, Eotaxin, IL-13, MIP-1α, IL-10, IL-1α, IP-10, GM-CSF and IFN-γ), 8 matrix metalloproteinases (MMP-1, -2, -3, -7, -8, -9, -10 and -13) and 10 soluble(s) factors (GP130, IL-2R, ICAM, E-selectin, Fas, TNF-R1, TNF-R2, RAGE, VCAM and MIF). The inter- and intra-assay variation was <10% (data not shown). The choice for the abovementioned biomolecules is motivated as follows. Inflammation caused by tissue damage (SIRS) is primarily mediated by activation of the inflammasome – consecutively raising IL-1β, TNF-α in the tissue. Theses cytokines cause the release of soluble TNF-receptors. In the plasma, soluble TNF-R2 appears to be more sensitive than sTNF-R1. In the infection-associated inflammation, TLR activation may be responsible for the induction of IFN-γ, as well as NF-kB-guided cytokines such as TNF-α, IL-8, IL-6, etc. some of which may be rapidly bound by receptors and are sensitive to detection assays using two monoclonal antibodies such as multiplexed bead associated assays. Generally, inflammation causes activation of many proteases and metalloproteases, which are either beneficial by degrading matrix proteins or are detrimental by cleaving receptors and contribute to anergy and non-responsiveness that appears to be a key factor in sepsis-associated pathogenesis. Finally, we chose soluble receptors to be determined since these biomarkers indicate which receptors are regulated in a defined disease state. Soluble iCAM, for instance, indicates whether transendothelial migration is one of the major processes going on at a defined time point. In a third aspect, we questioned the contribution of macrophages in the inflammatory process. Monocytes often disappear from the peripheral leukocyte population in patients with major SIRS or sepsis and we believe that they go to tissues. We chose MIP-1α to assess functional macrophage activity.

### Analysis methods

#### ANOVA analysis

We performed an ANOVA on each biomolecule to discover differences among the four disease codes (3-SIRS, 4-sepsis, 5-severe sepsis, 6-septic shock). The distributions of measured values were not close to a normal distribution (assumed by ANOVA), so the data were log transformed before analysis. The numbers of observations available varied due to different lengths of the patients' stay at the ICU. As shown in Table 1, some patients have many observation days with septic shock (such as patients #8 and #10), whereas others have observation days with SIRS only (patient #9). Four patients (#2, #5, #11 and #14) had days with SIRS and days with some form of sepsis, with patients #2, #5 and #14 first having some form of sepsis, and later have SIRS, and patient #11 starts with SIRS, which later turned into severe sepsis, and had SIRS again on the last day. Note that a patient can go from sepsis to SIRS if (s)he is successfully treated, as then microorganisms and infection may no longer be proven and hence the sepsis criteria are no longer met, while the criteria for SIRS can still be fulfilled.

**Table 1:** Observations contributed to each disease condition by each patient

Patient #	Days of disease condition (percentage of ICU stay)				
	
	3-SIRS	4-sepsis	5-severe sepsis	6-septic shock	Other/unknown
1	0 (0)	4 (40)	4 (40)	0 (0)	2 (20)
2	3 (38)	1 (12)	4 (50)	0 (0)	0 (0)
3	0 (0)	3 (43)	1 (14)	3 (43)	0 (0)
4	0 (0)	0 (0)	4 (29)	0 (0)	10 (71)
5	3 (43)	0 (0)	0 (0)	2 (29)	2 (29)
6	0 (0)	0 (0)	0 (0)	4 (100)	0 (0)
7	0 (0)	4 (80)	1 (20)	0 (0)	0 (0)
8	0 (0)	1 (10)	1 (10)	8 (80)	0 (0)
9	7 (100)	0 (0)	0 (0)	0 (0)	0 (0)
10	0 (0)	1 (7)	1 (7)	11 (86)	0 (0)
11	5 (71)	0 (0)	2 (29)	0 (0)	0 (0)
12	0 (0)	0 (0)	3 (27)	5 (45)	3 (27)
13	0 (0)	0 (0)	4 (50)	2 (25)	2 (25)
14	2 (50)	0 (0)	0 (0)	2 (50)	0 (0)
15	0 (0)	0 (0)	7 (58)	2 (17)	3 (25)
16	0 (0)	0 (0)	0 (0)	5 (100)	0 (0)
Observations	20	14	32	44	22
Patients	5	6	11	10	6

THE NUMBERS INDICATE DAYS OF DISEASE CONDITIONS. BETWEEN PARENTHESES IT IS GIVEN AS PERCENTAGE OF ICU STAY., SIRS - SYSTEMIC INFLAMMATORY RESPONSE SYNDROME

Unfortunately, no design could be found that permitted the rigorous separation of the patient and day effects. We therefore treated each observation as independent, but adjusted the degrees of freedom of the F test to correct for these effects. Bonferroni correction was used to correct for the multiple tests, as we test 28 biomolecules simultaneously.

#### Correlation analysis

Next, as a second step we performed a correlation analysis and computed the Pearson correlation between the log-transformed measurements of each of the biomolecules on the one hand, and each of the following eight labels on the other:

- SIRS (code 3) versus sepsis (code 4);
- SIRS (code 3) versus sepsis, severe sepsis and septic shock (codes 4, 5, 6);
- sepsis (code 4) versus severe sepsis and septic shock (codes 5, 6);
- sepsis (code 4) versus septic shock (code 6);
- sepsis (code 4) versus severe sepsis (code 5);
- the SOFA score;
- the SAPS II score and
- the outcome (survival/death).

As a reference, to determine the significance level for each of the computed correlations, we determined what correlations can be obtained by chance, by randomly permuting each label 10 000 times and computing the correlation to each of the biomolecules. In this way, we obtained expected correlations and variances, which we used to compute *P*-values. In addition, we again applied the Bonferroni-correction for multiple testing. Note again that the obtained *P*-values are biased, as we treated each sample as independent, whereas these come from a limited number of patients.

#### Pattern recognition analysis

In a third step, we performed a search for combinations of biomolecules that could give rise to a classifier to distinguish SIRS (code 3) from the septic condition (code 4). The idea was to see if a diagnostic biomarker set might be identified to distinguish sepsis from SIRS conditions. For this, a genetic algorithm[6] especially devised for subset selection was used. To identify small subsets of these biomolecules that would make the desired distinction, a support vector machine was trained on some of the observations and then tested on others. Because of missing data issues, six biomolecules were discarded (MMP-3, -7, IL-13, IFN-γ, sE-selectin and sVCAM). The final learning set consisted of 34 observations, 20 in SIRS and 14 in sepsis, from 10 patients among whom only patient #2 contributed observations to both conditions (see Table 1). Because of the stochastic nature of both the genetic search and the training of candidates on differing sets of observations, 10 independent searches were performed. Approximately two-thirds of the available observations were used for training and all were used for testing.

#### Prediction SIRS versus sepsis and predicting outcome

In a fourth step, we developed rules for predicting SIRS versus sepsis and for predicting mortality, and we generated the corresponding receiver operating characteristic (ROC) curves.

For predicting SIRS (code 3) versus sepsis (code 4), we took the individual biomolecules that we identified in the previous two steps, and simply used a threshold rule, i.e. above a certain threshold the patient's sample is classified as sepsis, and below it the sample is classified as SIRS. By varying the threshold, we created an ROC curve.

For predicting mortality, we applied a naïve Bayesian classifier,[7] using the panel of biomolecules that were identified to correlate significantly to the outcome. We applied a leave-one-out scheme, i.e., the classification of each sample is done by a classifier that is trained on the set of samples not including the particular sample at hand. By varying the classification threshold on the posterior probability, we again created an ROC curve. We also calculated sensitivity and specificity for the default threshold level and plotted the correctness of mortality prediction for the different patients and for their different days during the ICU stay. When making mortality predictions for new samples, the Bayesian classifier can be trained on all samples from this study, and directly applied to the measurements of the new samples.

#### Correlations between biomolecules

The final analysis was to compute the Pearson correlation between the log measurements for each pair of biomolecules.

## RESULTS AND DISCUSSION

### Results of the experiments

#### ANOVA analysis

**Figure 1:**
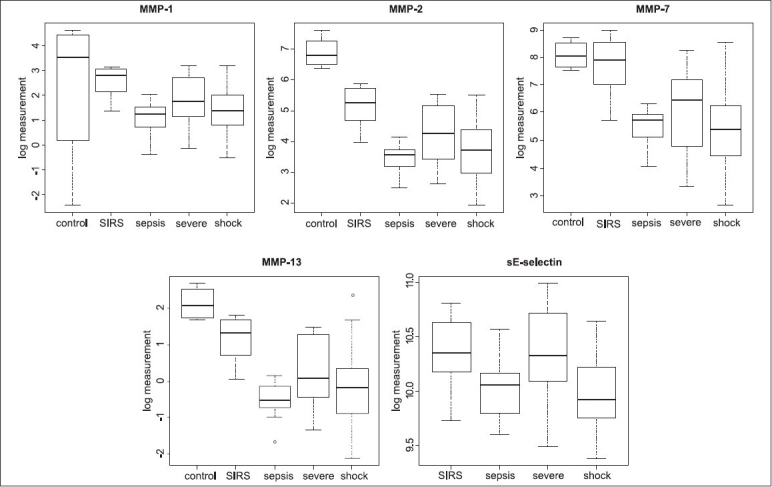
SIRS vs. sepsis biomarkers. Box plots show the distributions of the log measurements of plasma concentrations across the sample groups of the biomarkers MMP-1, -2, -7, -13 and soluble E-selectin, identified by the ANOVA and correlation analysis for the distinction between SIRS and sepsis

**Figure 2:**
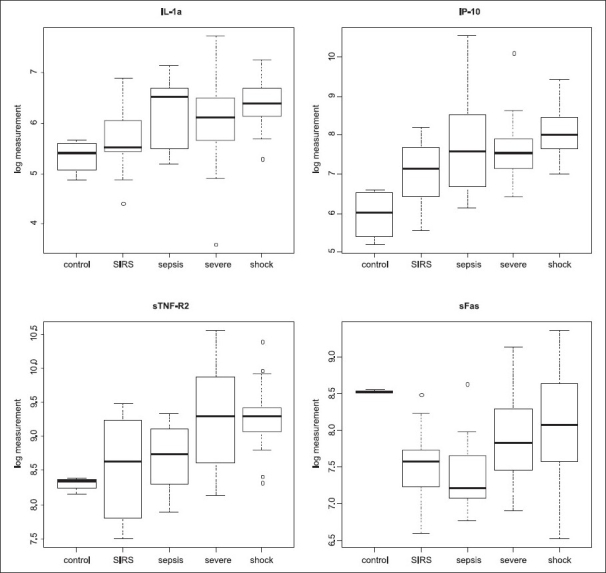
Additional sepsis severity biomarkers. Box plots show the distributions of the log measurements of plasma concentrations across the sample groups of the additional biomarkers IL-1α, IP-10, sTNF-R2 and sFas, identified by the ANOVA and correlation analysis for the distinction SIRS versus sepsis, severe sepsis and septic shock

The results of the ANOVA showed that MMP-1, -2, -7, -13 and sE-selectin are significantly lower in septic cases than in SIRS cases. Furthermore, IP-10, sFas and sTNF-R2 were elevated most in the plasma of patients in septic shock. Corresponding *P*-values after Bonferroni correction are below 0.01. We have to note, though, that these results may still contain the confounding of patients with sepsis state (see discussion). Figures 1 and 2 show the ranges of the log measurements for the above-described biomolecules.

#### Correlation analysis

The correlation analysis resulted in the following findings.

- MMP-1, -2, -7 and -13 plasma concentrations showed to be significantly lower (*P* < 0.001) in sepsis (code = 4) as compared to SIRS (code = 3), with absolute correlation values ∼0.8.
- In addition to these four, IL-1α, IP-10 and sTNF-R2 were higher (*P* < 0.05) in sepsis, severe sepsis and septic shock (codes = 4, 5, 6) as compared to SIRS (code = 3).
- sFas and sTNF-R2 were higher (*P* < 0.05) in severe sepsis and septic shock (codes = 5, 6) as compared to sepsis (code = 4) and also in septic shock (code = 6) as compared to sepsis (code = 4).
- MMP-2, MMP-7 and IL-1β were negatively correlated to the SAPS II score (*P* < 0.01) and MMP-3, IL-1α, IP-10, sFas, sTNF-R1 and sTNF-R2 were positively correlated to the SAPS II score (*P* < 0.001). The correlation of sTNF-R1 was relatively high (0.78).
- Many biomolecules were correlated with the SOFA score (*P* < 0.01), with however slightly lower absolute correlations (up to 0.50). MMP-2, -7, IL-1β, Eotaxin, GM-CSF and IFN-γ were negatively correlated, and MMP-3, -8, -10, IL-10, IP-10, sFas, sTNF-R1 and sTNF-R2 were positively correlated.
- The patients who died from septic complications had elevated levels of MMP-3, -10, IL-1α, IP-10, sIL-2R, sFas, sTNF-R1, sTNF-R2 and sRAGE as compared to the surviving patients (*P* < 0.05). In contrast, the former patients showed low levels of GM-CSF, IL-1β and Eotaxin (*P* < 0.05).

#### Pattern recognition analysis

Table 2 shows the set of best-performing classifiers. Each line in the table gives a biomarker set (consisting of two or three biomolecules), and the associated performance in terms of average number of errors over the 10 independent runs, and the maximum error over the 10 runs.

**Table 2:** Discovered biomarker sets that best distinguish SIRS condition from sepsis

Biomolecule sets			Mean error per search	
			
			Average	Worst
MMP-13	MIP-1α	sFas	1.381	1.500
MMP-13	MIP-1α	sgp130	1.431	1.522
MMP-13	MIP-1α	sICAM	1.160	1.314
MMP-13	MIP-1α	SIL-2R	1.341	1.639
MMP-13	MIP-1α	sRAGE	1.135	1.270
MMP-13	MIP-1α	sTNF-R2	1.249	1.338
MMP-2	MMP-8		1.329	1.421
MMP-2	MMP-8	sgp130	1.425	1.722
MMP-2	MMP-8	sICAM	1.494	1.923
MMP-2	MMP-8	MIP-1α	1.314	1.571
MMP-2	MMP-8	MMP-13	1.120	1.147
MMP-2	MMP-8	sRAGE	1.544	1.891
MMP-8	MMP-13	MMP-10	1.378	1.564
MMP-8	MMP-13		1.122	1.235
MMP-8	MMP-13	sFas	1.222	1.377
MMP-8	MMP-13	sgp130	1.030	1.104
MMP-8	MMP-13	sICAM	1.225	1.291
MMP-8	MMP-13	sIL-2R	1.318	1.396
MMP-8	MMP-13	MIP-1α	1.012	1.153
MMP-8	MMP-13	sRAGE	1.067	1.146
MMP-8	MMP-13	sTNF-R1	1.389	1.653
MMP-8	MMP-13	sTNF-R2	1.149	1.271
MMP-8	MMP-13	MMP-9	1.420	1.532
MMP-9	MMP-13	MIP-1α	1.395	1.488

We note that in no case did any of these classifiers make an average (over the 10 runs) worse than 2 errors out of a possible 34. We see the MMPs are strongly being represented. Half of the errors are contributed by measurements of patient #11 on day 8, while the other half is contributed by patient #2, the only patient contributing measurements to both the SIRS and sepsis classes. This may be an indication of the possible confounding of patient effects with the clinical distinction we are seeking.

**Figure 3:**
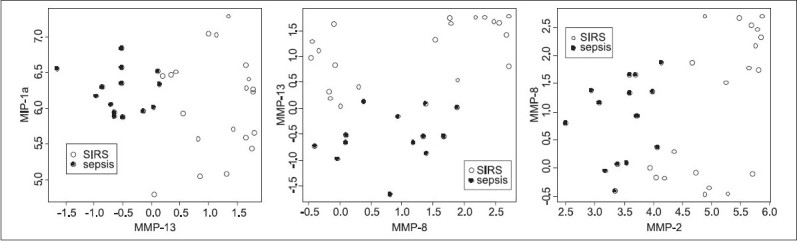
Best-separating biomarker pairs. Biplots are shown for the three pairs of biomolecules that occur most in the sets that performed well on classifying between SIRS and sepsis

Table 2 indicates three strong pairs of compounds: MMP-2 with MMP-8, MMP-8 with MMP-13 and MMP-13 with MIP-1α (although this pair did not meet our error cutoff without adding a third biomolecule). The role of MIP-1α and MMP-8 is quite striking, as they are in themselves hardly indicative for being SIRS (code 3) or sepsis (code 4); the correlation analysis indicated that their correlations to that label are quite weak. To get more insight, we plotted the log measurements for samples with codes 3 and 4, for each of these three pairs of compounds; see Figure 3. As we can see in the figure, the main separator is MMP-13 in the left and middle graph, and MMP-2 in the right hand graph. MIP-1α and MMP-8 seem to make the separation only slightly better.

#### Prediction SIRS versus sepsis and predicting outcome

**Figure 4:**
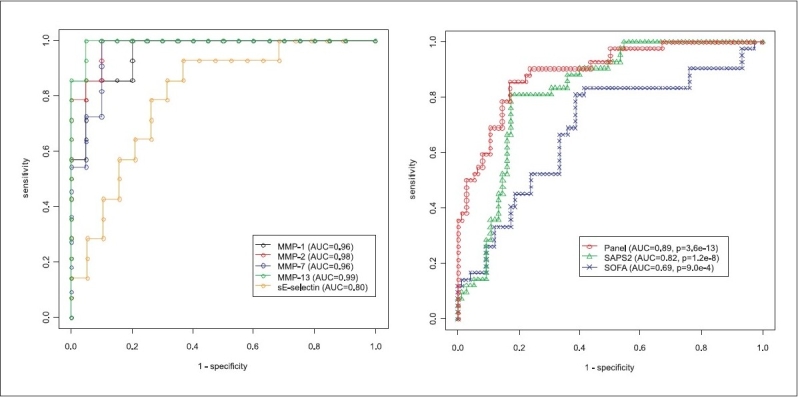
ROC curves. The left figure shows an ROC curve for the distinction between SIRS and sepsis, for the five most-distinguishing biomarkers. The right figure shows an ROC curve for predicting mortality, using the SAPS II score, the SOFA score and the identified panel of 12 biomarkers (MMP-3, -10, IL-1α, IP-10, sIL-2R, sFas, sTNF-R1, sTNF-R2, sRAGE, GM-CSF, IL-1β and Eotaxin)

The ROC curves are shown in Figure 4. We see that SIRS can be distinguished from sepsis by using MMP-1, -2, -7, or -13 with an AUC of more than 0.95 (*P* < 2e-6); sE-selectin performs a bit worse, with an AUC of 0.80 (*P* < 0.005). For predicting mortality, we see that by using the panel of biomolecules identified by the correlation analysis, consisting of MMP-3, -10, IL-1α, IP-10, sIL-2R, sFas, sTNF-R1, sTNF-R2, sRAGE, GM-CSF, IL-1β and Eotaxin, we get a much higher AUC (0.89, *P* = 3.6e-13) than by using the SAPS II or SOFA score.

**Figure 5:**
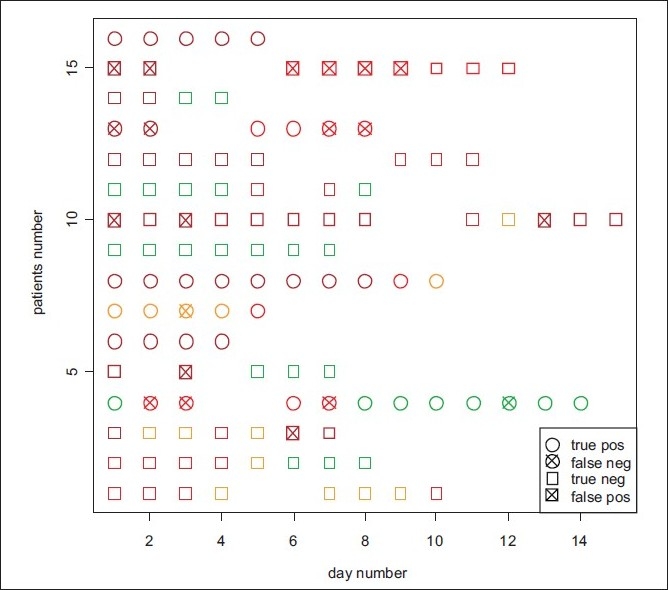
Correctness of predicting mortality. For each patient and each of the days during his/her ICU stay it is indicated whether mortality is correctly predicted, for a Bayesian classifier using the identified panel of 12 biomarkers (MMP-3, -10, IL-1α, IP-10, sIL-2R, sFas, sTNF-R1, sTNF-R2, sRAGE, GM-CSF, IL-1β and Eotaxin). The colors indicate the sepsis score (green: score 3 - SIRS or less; orange: score 4 - sepsis; red: score 5 - severe sepsis; dark red: score 6 - septic shock)

Figure 5 shows the correctness of the mortality prediction for all patients and all their days during his/her ICU stay. We observe that the predictions are already accurate during the first two days for 12 of the 16 patients (although patient #5 has no measurement on day two), two patients (#4 and #10) have a wrong prediction on one of the first two days, and two patients (#13 and #15) have wrong predictions on their both first days. This means that the classifier can be used as an early predictor of mortality. Counting the overall samples, we have 33 true positive and 9 false negative predictions, giving a sensitivity of 79%. Furthermore, we have 65 true negative and 11 false positive predictions, giving a specificity of 86%.

#### Correlations between biomolecules

**Figure 6:**
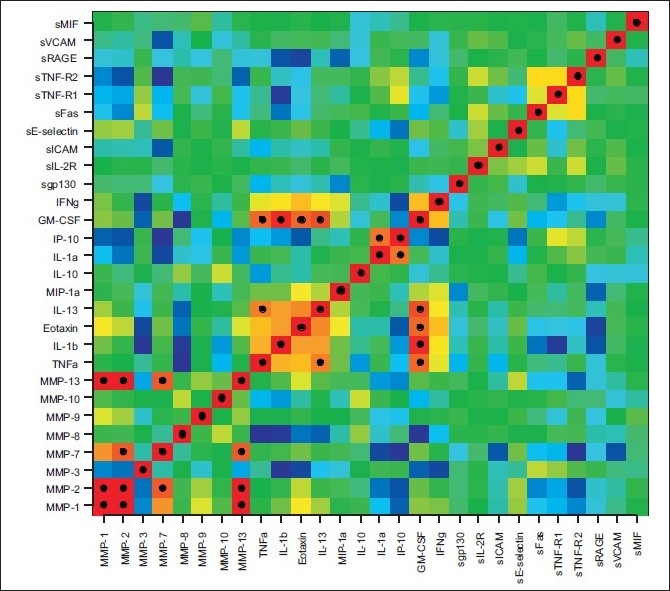
Correlation plot of the 28 measured biomolecules. Shown is the Pearson correlation between the log measurements of each pair of biomolecules. Dark red means a correlation of +1 (see e.g., the diagonal), and dark blue means a correlation of −1. Furthermore, black dots indicate correlations with an absolute value greater than 0.8

The Pearson correlation between the log measurements for each pair of biomolecules is depicted in Figure 6. Three groups of strongly correlating biomarker concentrations appear in the figure:

- MMP-1, MMP-2, MMP-7 and MMP-13;
- TNF-α, IL-1β, Eotaxin, IL-13 and GM-CSF and
- IL-1α and IP-10.

### Biological interpretation

#### MMPs and sE-selectin

When we combined biomolecules that could give rise to a better distinction of SIRS and sepsis, using a genetic algorithm devised for subset selection, we found the following remarkable biomarker pairs: MMP-13 with MIP-1α (not alone, but combined with either sFas, sGP-130, sICAM, sIL-2R, sRAGE or sTNF-R2), MMP-2 with MMP-8 and MMP-8 with MMP-13. All of these are related to tissue remodeling and leukocyte chemotaxis and recruitment. MMP-2 and -13 are also best classifiers for SIRS and sepsis as demonstrated in the biplot in Figure 3 and the ROC curves in Figure 4. Correlation analysis [Figure 6] shows that MMP-1, -2, -7 and -13 strongly correlate. Out of the inflammatory cytokine panel IL-1β and TNF-α correlated, which supports the observation that TNF-α is a major inducer of IL-1β in sepsis, which has been known for a long time.[8] Eotaxin, IL-13 and GM-CSF may play a role in concomitant recruitment of TH2 lymphocytes and myeloid progenitors including eosinophils, respectively. The correlation of IL-1α and IP-10 detected here is indeed challenging since the chemoattractant IP-10 is dependent on Interferon-activation and IL-1α is a member of the highly promiscuous cytokine family related to NF-kB activation. The biomarkers identified are interesting because of arguments based on clinical observations but also based on basic science.

In this study, we provide evidence for a high discriminative value of defined MMPs between SIRS (code = 3) and sepsis (code = 4). Following Figure 1 and the ANOVA and correlation analyses, MMP-1, -2, -7, -13 and sE-selectin are higher in SIRS as compared to sepsis. MMP-7 remains low in all stages of sepsis including septic shock. This MMP-7 was found to be significantly lower in patients with an NQO-1 mutation suggesting that MMP-7 diminution may be related to oxidative stress (Schneider *et al*., unpublished data). Matrix metalloproteinases are involved in extracellular matrix remodeling and cell migration that occur following injury or infections. MMP-7 mediates cleavage of E-cadherin to stimulate transepithelial migration of neutrophils controlling bacterial infections. Studies in mice suggest that MMP-7 also regulates the antimicrobial activity of defensins in intestinal mucosa.[9] Moreover, MMP-7, the matrilysin cleaves a number of nonstructural proteins, such as pro-TNF-α and Fas-L,[10] and contributes to the pool of active cytokines and soluble receptors. MMP-1 has been demonstrated to activate MMP-3 and stimulate tumor cell migration.[11] A previous study by Hoffmann *et al*. found no differences in the plasma levels of MMP-2 between healthy controls and septic patients.[12] Others found that MMP-2, a type IV collagenase, is present in joints and plasma of healthy controls but is activated by thrombin,[13] suggesting that sepsis-associated decline of MMP-2 may be due to binding of selective inhibitors or transcriptional downregulation. The patterns of MMP-1, -2 and -13 are very similar suggesting that these enzymes play a major role in stress-induced tissue remodeling.[14] Although MMP-9 and -13 may correlate in other studies, we did not find significant differences in the MMP-9 protein levels between SIRS and septic patients. Studies using MMP-9 gene deficient mice (MMP-9-/-) and normal wild-type mice both infected (i.p) with Escherichia coli showed that MMP-9 deficient mice displayed a reduced resistance against E. coli, as indicated by an enhanced bacterial outgrowth in the peritoneal cavity and increased dissemination of infection.[15]

Next to the decreased levels of MMP in septic patients, we also found decreased levels of sE-selectin. E-selectin is an early mediator of leukocyte-endothelial adhesion, and is expressed on activated endothelium.[16] In our study, we observed a relatively low E-selectin secretion into the blood stream as the diseases progressed from SIRS to sepsis. The vascular endothelium controls leukocyte extravasation into tissue by the induction and modulation of endothelial cell adhesion molecules, such as E-selectin (CD62E). E-selectin is not expressed by non-stimulated endothelium, but is activated by cytokines and initiates neutrophil recruitment in sepsis-induced lung injury.[17] According to our results, soluble E-selectin is an important parameter in the transition of SIRS and sepsis. Specifically, sE-selectin has not been found to increase with the manifestation of sepsis; it increases in a longitudinal course during severe sepsis. The significance of sE-selectins and sICAM-1 levels during severe acute stress in children has been described earlier.[18] Cummings *et al*.[16] studied septic patients as compared to critically ill medical ICU patients and found an increase of sE-selectin plasma concentrations in sepsis. Unfortunately, in our study a distinction between sepsis and severe sepsis has not been made [Figure 1]. The age-matched individuals studied as a control population had elevated sE-selectin concentrations as well; however, these individuals were studied pre-operatively. Results indicate that biomarkers need to be generally studied in a defined clinical context and may not be specific for a defined disease.

#### Cytokines

Plasma protein concentrations of IL-1α, IP-10 and sTNF-R2 were higher in septic patients compared to SIRS. Moreover, these markers, together with sFas were even more elevated in patients with septic shock [Figure 2] suggesting that these biomarkers correlate with staging septic patients and intensive care. In a study evaluating key cytokines and chemokines in preterm infants, IP-10 with a plasma cutoff concentration ≥1250 pg/mL could identify all septemic infants with the highest sensitivity (93%) and specificity (89%) at 0 h.[19] IL-1α is a pleiotropic cytokine related to inflammation and tissue injury.[20] IP-10 is a chemokine, CXCL10, secreted by TH1 lymphocytes upon IFN-γ stimulation.[21] Binding to CXCR3, IP-10 induces recruitment of T-cells,[22] but also natural killer effectors and dendritic cells. Moreover, IP-10 is a new member of LPS-responsive cytokines.[23] Recently, the application of IP-10 specific antibodies has been suggested to cure Type I diabetes.[24] As exemplified by CCR4 deficiency, chemokine receptor deficiency related to leukocyte recruitment may result in improved survival following septicemia.[25,26] Upon progression of sepsis, we observed elevated plasma protein concentrations of sTNF-R2 and sFas (sCD95). Both mediators were correlated to disease severity. In the case of CD95, these results may mean that more CD95 is expressed and concomitant to increased protease activity, a greater amount of soluble receptor is expected to be released into the circulation. Soluble TNF receptors are well-recognized to indicate the severity of organ failure[27] and indicate the immune system's attempt to limit pro-inflammation. In patients with sepsis and acute hepatic failure, a correlation between TNF-α and sFas has been shown.[28] The same study suggested that sFas-ligand was protective. The selective elevation of sFas in patients with sepsis as compared to SIRS has been shown in adults[29] and with an even greater impact in pediatric cases substantiating a role for sFas in inflammatory deregulation in severe sepsis.[30] In another study, it has been demonstrated that the onset of severe sepsis and macrophage apoptosis was rather related to mitochondrial dysfunction and Hsp70 rather than soluble Fas or Bcl-2.[31] It may be concluded that the results of sFas are directed to organ injury rather than apoptosis of circulating leukocytes. Indeed, tissue and organ damage leading to multiple organ dysfunctions and death constitute end-stage parameters in a lethal course of sepsis.

### Strengths, limitations and future directions

The strengths of the analysis presented in this paper is to provide information on a reasonably small set of biomarkers selected from the huge amount of cytokines, chemokines and molecules, which are released upon inflammation. This set of markers bears some that correlate and others that cooperate to define the severity of disease. Given a certain time point of a patient's course, the designation of clinical scores for sepsis, SOFA and SAPS II have been established to predict outcome. Unfortunately, none of these scores include immune markers, despite the fact that patients under intensive care die from immune deficiency and anergy, which classically follow hyper-inflammation.

As exemplified here, the state of blood infections cannot be deduced from scores, however, it can be from immune profiling (see patients #2, #5, #11 and #14). Since we also included markers indicative for oxidative stress that may have accumulated already before major trauma, such as sRAGE, the current strategy implements a pre-trauma risk status.

It may be regarded as a limitation, but it may also be an advantage to include several observations of a single patient's course as independent event since chronicity of anergy is a characteristic of sepsis and a more dynamic behavior is a characteristic of SIRS.

A major limitation of our current approach remains to be the significance of the biosignature identified. A small number of patients in a fairly homogenous clinical setting has been followed so far, for practical reasons. More patients would make such a study to become even more laborious, time consuming and expensive.

Future directions of biosignatures are to support the predictive value of currently applied clinical scores. We foresee a comprehensive discussion on individual molecules such as sE-selectin or metalloproteinases to understand the regulation of hyper-inflammation and non-responsiveness. Although not yet reported for SIRS and sepsis, the most recent relationship between myeloid-derived suppressor cells and metalloproteinases may be used as a hint into the right direction.[32]

## CONCLUSION

In conclusion, the current analysis stresses the relevance of MMP-1, -2, -7, -13 and sE-selectin concentrations for the transition of SIRS to sepsis. The down-modulation of MMPs may be relevant to facilitate receptor re-expression, which was impaired in an infection-independent systemic inflammation (SIRS). Further, we identified a set of biomarkers to distinguish between SIRS, sepsis, severe sepsis and septic shock. These were IL-1α related to apoptosis and inflammation, IP-10 related to leukocyte recruitment into inflamed organs, sTNF-R2 and sFas related to a pro-inflammatory environment with little effect on a beneficial functional modulation of the sepsis scenario. The feasibility of multiplex-guided determinations accompanied by documentation of simplified staging may be suitable to apply in a validation study with a large number of patients. Most importantly, the functional relevance of biomarkers identified may be promising in designing therapeutic interventions to influence the course of disease.

## References

[1] (1992). American College of Chest Physicians/Society of Critical Care Medicine Consensus Conference: definitions for sepsis and organ failure and guidelines for the use of innovative therapies in sepsis. Crit Care Med.

[2] Angus DC, Linde-Zwirble WT, Lidicker J, Clermont G, Carcillo J, Pinsky MR (2001). Epidemiology of severe sepsis in the United States: Analysis of incidence, outcome, and associated costs of care. Crit Care Med.

[3] Hotchkiss RS, Karl IE (2003). The pathophysiology and treatment of sepsis. N Engl J Med.

[4] Le Gall JR, Lemeshow S, Saulnier F (1993). A new Simplified Acute Physiology Score (SAPS II) based on a European/North American multicenter study. JAMA.

[5] Vincent JL, Moreno R, Takala J, Willatts S, De Mendonça A, Bruining H (1996). The SOFA (Sepsis-related Organ Failure Assessment) score to describe organ dysfunction/failure. On behalf of the Working Group on Sepsis-Related Problems of the European Society of Intensive Care Medicine. Intensive Care Med.

[6] Schaffer JD, Janevski A, Simpson M (2005). A genetic algorithm approach for discovering diagnostic patterns in molecular measurement data. Proc. CIBCB.

[7] Duda RO, Hart PE, Stork DG (2001). Pattern Classification.

[8] Tracey KJ, Cerami A (1990). Metabolic responses to cachectin/TNF. A brief review. Ann N Y Acad Sci.

[9] Ayabe T, Satchell DP, Pesendorfer P, Tanabe H, Wilson CL, Hagen SJ, Ouellette AJ (2002). Activation of Paneth cell alpha-defensins in mouse small intestine. J Biol Chem.

[10] Somerville RP, Oblander SA, Apte SS (2003). Matrix metalloproteinases: Old dogs with new tricks. Genome Biol.

[11] Benbow U, Schoenermark MP, Mitchell TI, Rutter JL, Shimokawa K, Nagase H (1999). A novel host/tumor cell interaction activates matrix metalloproteinase 1 and mediates invasion through type I collagen. J Biol Chem.

[12] Hoffmann U, Bertsch T, Dvortsak E, Liebetrau C, Lang S, Liebe V (2006). Matrix-metalloproteinases and their inhibitors are elevated in severe sepsis: Prognostic value of TIMP-1 in severe sepsis. Scand J Infect Dis.

[13] Sharony R, Pintucci G, Saunders PC, Grossi EA, Baumann FG, Galloway AC (2006). Matrix metalloproteinase expression in vein grafts: Role of inflammatory mediators and extracellular signal-regulated kinases-1 and -2. Am J Physiol Heart Circ Physiol.

[14] Henderson BC, Sen U, Reynolds C, Moshal KS, Ovechkin A, Tyagi N (2007). Reversal of systemic hypertension-associated cardiac remodeling in chronic pressure overload myocardium by ciglitazone. Int J Biol Sci.

[15] Renckens R, Roelofs JJ, Florquin S, de Vos AF, Lijnen HR, van't Veer C (2006). Matrix metalloproteinase-9 deficiency impairs host defense against abdominal sepsis. J Immunol.

[16] Cummings CJ, Sessler CN, Beall LD, Fisher BJ, Best AM, Fowler AA (1997). Soluble E-selectin levels in sepsis and critical illness. Correlation with infection and hemodynamic dysfunction. Am J Respir Crit Care Med.

[17] Tsokos M, Fehlauer F, Puschel K (2000). Immunohistochemical expression of E-selectin in sepsis-induced lung injury. Int J Legal Med.

[18] Briassoulis G, Papassotiriou I, Mavrikiou M, Lazaropoulou C, Margeli A (2007). Longitudinal course and clinical significance of TGF-beta1, sL- and sE-Selectins and sICAM-1 levels during severe acute stress in children. Clin Biochem.

[19] Ng PC, Li K, Chui KM, Leung TF, Wong RP, Chu WC (2007). IP-10 is an early diagnostic marker for identification of late-onset bacterial infection in preterm infants. Pedaitr Res.

[20] Shindo S, Ogata K, Kubota K, Kojima A, Kobayashi M, Tada Y (2003). Vascular prosthetic implantation is associated with prolonged inflammation following aortic aneurysm surgery. J Artif Organs.

[21] Luster AD, Unkeless JC, Ravetch JV (1985). Gamma-interferon transcriptionally regulates an early-response gene containing homology to platelet proteins. Nature.

[22] Panzer U, Steinmetz OM, Paust HJ, Meyer-Schwesinger C, Peters A, Turner JE (2007). Chemokine receptor CXCR3 mediates T cell recruitment and tissue injury in nephrotoxic nephritis in mice. J Am Soc Nephrol.

[23] Ovstebo R, Olstad OK, Brusletto B, Moller AS, Aase A, Haug KB (2008). Identification of genes particularly sensitive to lipopolysaccharide (LPS) in human monocytes induced by wild-type versus LPS-deficient Neisseria meningitidis strains. Infect Immun.

[24] Ejrnaes M, von Herrath MG, Christen U (2006). Cure of chronic viral infection and virus-induced type 1 diabetes by neutralizing antibodies. Clin Dev Immunol.

[25] Chvatchko Y, Hoogewerf AJ, Meyer A, Alouani S, Juillard P, Buser R (2000). A key role for CC chemokine receptor 4 in lipopolysaccharide-induced endotoxic shock. J Exp Med.

[26] Ness TL, Ewing JL, Hogaboam CM, Kunkel SL (2006). CCR4 is a key modulator of innate immune responses. J Immunol.

[27] Iglesias J, Marik PE, Levine JS (2003). Elevated serum levels of the type I and type II receptors for tumor necrosis factor-alpha as predictive factors for ARF in patients with septic shock. Am J Kidney Dis.

[28] Nakae H, Narita K, Endo S (2001). Soluble Fas and soluble Fas ligand levels in patients with acute hepatic failure. J Crit Care.

[29] Torre D, Tambini R, Manfredi M, Mangani V, Livi P, Maldifassi V (2003). Circulating levels of FAS/APO-1 in patients with the systemic inflammatory response syndrome. Diagn Microbiol Infect Dis.

[30] Doughty L, Clark RS, Kaplan SS, Sasser H, Carcillo J (2002). sFas and sFas ligand and pediatric sepsis-induced multiple organ failure syndrome. Pediatr Res.

[31] Adrie C, Bachelet M, Vayssier-Taussat M, Russo-Marie F, Bouchaert I, Adib-Conquy M (2001). Mitochondrial membrane potential and apoptosis peripheral blood monocytes in severe human sepsis. Am J Respir Crit Care Med.

[32] Melani C, Sangaletti S, Barazzetta FM, Werb Z, Colombo MP (2007). Amino-biphosphonate-mediated MMP-9 inhibition breaks the tumor-bone marrow axis responsible for myeloid-derived suppressor cell expansion and macrophage infiltration in tumor stroma. Cancer Res.

